# Agreement between ambulance paramedics and physicians on the (pre)hospital identification of low-risk chest pain patients

**DOI:** 10.1097/MEJ.0000000000001274

**Published:** 2025-12-22

**Authors:** Cyril Camaro, Goaris W.A. Aarts, Marc A. Brouwer, Sjoerd W. Westra, Niels van Royen

**Affiliations:** Department of Cardiology, Radboud University Medical Centre, Nijmegen, the Netherlands

Patients with suspected non-ST-segment elevation acute coronary syndrome (NSTE-ACS) are routinely transported to the emergency department (ED) by ambulance [1]. However, in the majority of the suspected NSTE-ACS patients, no acute coronary syndrome (ACS) is found [2,3]. Identification of patients who are not likely to have an ACS is possible with the HEART (History, ECG, Age, Risk factors, and Troponin) score, which is a widely validated risk stratification tool for chest pain patients at the ED [4]. Implementing a point-of-care (POC) troponin measurement enables ambulance paramedics to assess the HEART score in the prehospital setting [5,6]. To widely implement such a strategy of prehospital rule-out, a correct low-risk classification (HEART score of ≤3) is essential.

In this prespecified subanalysis of the ARTICA (Acute Rule out of non ST-segment elevation acute coronary syndrome in the (pre)hospital setting bij HEART score assessment and a single poInt of CAre troponin) trial, we evaluated the level of agreement between ambulance paramedics and physicians regarding the classification of patients as low-risk, within a prehospital population deemed eligible for early rule-out of NSTE-ACS. The ARTICA trial is a randomised trial on prehospital rule-out of NSTE-ACS in low-risk patients using a POC troponin measurement, and primary results and design were published previously [7,8].

Suspected NSTE-ACS patients ≥ 18 years, with a low prehospital HEAR score (≤3) were included in the current study if they were transported to the hospital as part of the ARTICA trial design and if data were available to assess an in-hospital HEAR score. Assessment of the HEAR score by ambulance paramedics was performed on-site. The in-hospital HEAR score was retrospectively assessed by two independent physicians. The prehospital and in-hospital HEAR scores were compared, and the intraclass correlation coefficient (ICC) was calculated.

A total of 388 patients were included. The mean age was 53.1 (±12.4) years and 56.2% were female. Agreement on the low-risk classification (HEAR score of ≤3) was achieved in 88.9%. The prehospital total HEAR score was identical to the in-hospital HEAR score in 152 patients (39.2%), lower in 86 (22.2%), and higher in 150 (38.7%). The overall ICC was 0.67 [95% confidence interval (CI): 0.59–0.73]. Prehospital History scores were significantly higher and prehospital ECG scores were significantly lower. The mean prehospital History score was significantly higher than the mean in-hospital History score, with a mean difference of +0.35 (95% CI: 0.28–0.42, *P* < 0.001). The mean prehospital ECG score was significantly lower than the mean in-hospital ECG score, with a mean difference of −0.19 (95% CI: −0.24 to −0.15, *P* < 0.001) (Fig. 1).

**Fig. 1:**
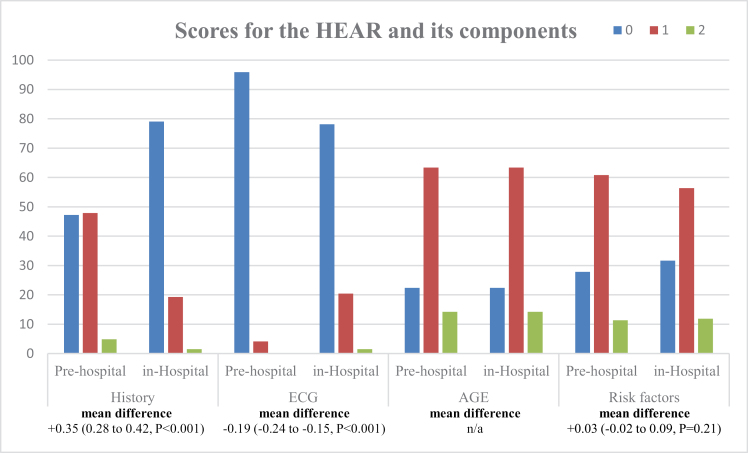
Scores for the HEAR score and its components. HEAR, History, ECG, Age, and Risk factors.

This study demonstrated that physicians in the hospital agreed with ambulance paramedics on a low-risk classification (HEAR score ≤3) for NSTE-ACS in a high percentage (88.9%). The mean HEAR score was similar. Overall agreement of the HEAR score, as assessed by physicians in-hospital and ambulance paramedics prehospital, was moderate with an ICC of 0.66. This was primarily influenced by the history and ECG components, which yielded the lowest ICC values. Only 39.2% of patients that had exactly identical prehospital and in-hospital HEAR scores (Table 1).

**Table 1:** Differences in the assessment of the History, ECG, Age, and Risk factors score and its components

	History	ECG	Age	Risk factors	HEAR score
Prehospital lower	31 (8.0%)	77 (19.8%)	0	44 (11.3%)	86 (22.2%)
Equal	200 (51.5%)	304 (78.4%)	388 (100.0%)	287 (74.0%)	152 (39.2%)
Prehospital higher	157 (40.5%)	7 (1.8%)	0	57 (14.7%)	150 (38.7%)

HEAR, History, ECG, Age, and Risk factors.

Of the 388 patients identified as low-risk patients by ambulance paramedics, 43 (11%) had an in-hospital HEAR score of >3. In these patients, the underestimation of the HEAR score was mostly caused by an underestimation of the ECG component. In the group with disagreement regarding low-risk classification, the incidence of MACE was significantly higher compared to the group with consensus on low-risk classification. However, five patients in the disagreement group had elevated troponin levels at first presentation at the ED, which could also have been addressed through prehospital POC troponin analysis.

More correct assessment of the ECG component can probably be achieved by transmission of the ECG to a hospital if POC troponin measurement to rule-out an NSTE-ACS is being considered. Also, adding a POC troponin increases agreement between prehospital and in-hospital risk classification. Finally, the assessment of ECGs using artificial intelligence algorithms is expected to advance significantly in the near future.

Limitations include the retrospective calculation based on the medical records and in contrast to the ambulance paramedics on-site, the physicians at the ED had access to the more detailed medical history of the patients. Moreover, the treating physicians at the ED had more information about the course of the symptoms, the reaction to sublingual nitrates and troponin levels.

In summary, in a population of prehospital identified low-risk patients, physicians in the hospital agreed with the ambulance paramedics on the low-risk classification in a high percentage of patients. Continuous history training and multidisciplinary ECG assessments are strongly recommended to further enhance the prehospital use of the HEAR score.

## Acknowledgements

The ARTICA trial was supported by a grant from the Netherlands Organisation for Health Research and Development (ZonMw), 2018 grant number 850001942. ZonMw had no role in the design or monitoring of the trial, the enrolment of participants, the collection, storage, retention or analysis of the data, the writing of the manuscript or the decision to submit the manuscript for publication.

### Conflicts of interest

C.C. reports a grant from the Netherlands Organisation for Health Research and Development (ZonMw) and consulting fees from AstraZeneca. N.v.R. reports grants from Abbott, Philips, Medtronic, and Biotronik and speaker fees from Microport, Abbott, Rainmed, and Bayer. The other authors have no conflicts of interest.
